# Interpartner Agreement on Intimate Partner Violence Reports: Evidence From a Community Sample of Different-Sex Couples

**DOI:** 10.1177/10731911231196483

**Published:** 2023-09-21

**Authors:** Marta Capinha, Daniel Rijo, Marlene Matos, Marco Pereira

**Affiliations:** 1 Faculty of Psychology and Educational Sciences, Center for Research in Neuropsychology and Cognitive and Behavioral Intervention, University of Coimbra, Coimbra, Portugal; 2 Psychology Research Center, School of Psychology, University of Minho, Braga, Portugal

**Keywords:** interpartner agreement, inter-rater reliability, intimate partner violence, Gwet’s AC1, intra class correlation, proxy method, revised Conflict Tactic Scales

## Abstract

An accurate assessment of intimate partner violence (IPV) is crucial to guide public policy and intervention. The Conflict Tactic Scales Revised (CTS-2) is one of the most widely used instruments to do so. Despite its good psychometric properties, research on interpartner agreement has pointed to low-to-moderate estimates, which generated some concerns about the validity of the results obtained through single-partner reports. This cross-sectional study introduces indexes that have not previously been used to assess interpartner agreement. Both partners’ reports on perpetration and victimization were analyzed in a community sample of 268 different-sex couples. Our results generally pointed to better agreement levels on IPV occurrence than frequency, suggesting that the proxy method (i.e., using a single-partner report) could be a reliable method for assessing IPV occurrence but not its frequency in this population. Findings are discussed as well as the advantages and constraints of different IPV assessment practices.

Intimate partner violence (IPV) is a concerning and highly prevalent social problem (e.g., Capinha et al., 2022; Esquivel-Santoveña et al., 2013; European Union Agency for Fundamental Rights [FRA], 2014), with severe and intergenerational consequences for those involved (e.g., Coker et al., 2002; Miller & McCaw, 2019; Romano et al., 2021). Although IPV is a relational phenomenon, research on the topic has mainly relied on data from only one of the partners (also called the proxy method; Armstrong et al., 2002). This method is usually used in studies about the prevalence (i.e., occurrence) of IPV and its correlates (e.g., Occean et al., 2021; Ruiz-Pérez et al., 2017), including those of the World Health Organization (WHO) focusing on women’s victimization in intimate relationships (e.g., Garcia-Moreno et al., 2006; World Health Organization [WHO], 2017). Difficulties in assessing both partners (Straus et al., 1996), including costs (Moffitt et al., 1997), have posed obstacles to couples’ assessment, especially in large-scale or epidemiological studies.

However, assessing only one partner may introduce biases, and relevant questions remain regarding whether a single element of the couple can provide valid reports of aggressive behaviors in the relationship. If that is not the case, the use of a proxy method (i.e., using a single-partner report) may lead to different findings depending on the use of men’s or women’s reports. This is particularly critical as the self-report of bidirectional aggression (i.e., perpetration of at least one form of aggressive behavior by each partner), and the symmetry in prevalence rates (i.e., similar rates of IPV occurrence toward men and women) have been widely identified across different countries (for reviews see Esquivel-Santoveña et al., 2013; Langhinrichsen-Rohling et al., 2012) but still disputed by some authors (e.g., Kang et al., 2017; Wood, 2015). Therefore, establishing the proxy method as reliable when assessing IPV would be essential to ensure that the results of studies using this method are trustworthy.

## The Assessment of Interpartner Agreement on IPV

As the actual occurrence (i.e., presence or absence of the behavior) and frequency (i.e., number of times a behavior occurred) of IPV behaviors within couples are difficult to verify through other means (e.g., observation), research has largely depended on self-report measures. In this context, inter-rater reliability has received increased attention as a way to estimate the reliability of these instruments and to discuss findings that rely on the proxy method (Vega & O’Leary, 2007). In the case of IPV, interrater reliability can be measured through interpartner agreement. In particular, the more frequently partners agree on their reports of IPV, the more the IPV scores they assign are considered reliable (Gwet, 2014). Therefore, efforts have been made to understand whether the self-report of IPV perpetration by one partner is similar to the self-report of IPV victimization by the other partner. The present work contributes to those efforts, exploring interpartner agreement through indexes whose use is a novelty in this field of research.

In a literature review, Armstrong et al. (2002) claimed that only five of the 15 reviewed studies have found “some level” (p. 9) of interpartner agreement in heterosexual couples and concluded that using the proxy method cannot reliably assess IPV. Most of the reviewed research used the original version of the Conflict Tactic Scale (CTS; Straus, 1979) or some modified version, and most relied on the percentage of agreement/disagreement. The authors recommended that further research exploring IPV interpartner agreement should use more than one agreement index, as findings may vary with different indexes. More recent research, using different instruments in different countries (e.g., Marshall et al., 2021; Riesgo González et al., 2019; Strandmoen et al., 2016; Yoshikawa et al., 2021) has improved on the limitations of previous studies by resorting to various index agreements. Nonetheless, these studies found low-to-moderate agreement between partners’ reports. The use of different instruments impaired further conclusions about the reliability of using one partner versus both partners reports because findings might also reflect issues related to the instruments themselves.

Given the findings described earlier, most authors continued to recommend using different indexes when assessing agreement and collecting both partners’ reports of IPV (e.g., O’Leary & Williams, 2006; Yoshikawa et al., 2021). The use of both partners’ reports has known advantages compared with the proxy method. In particular, it provides a broader knowledge of each partner’s views and even the couple’s dynamics. However, such a strategy is not always viable or cost-effective. Although research has shown that interpartner agreement regarding other behaviors (that are not IPV or socially undesirable) also ranges from low to moderate (Marshall et al., 2011; O’Leary & Williams, 2006), the (dis)agreement between partners’ reports remains an important concern for both researchers and practitioners. For example, some authors warned that IPV reports are particularly prone to social desirability (Moffitt et al., 1997) and are influenced by factors that contribute to discrepancies between partners’ reports in any field (e.g., memory, education, shame, and measurement error; Armstrong et al., 2002; Marshall et al., 2011; Moffitt et al., 1997; Yoshikawa et al., 2021). Moreover, the fact that one might fail to recognize himself/herself as a victim or a perpetrator of IPV has added more concerns regarding assessing this phenomenon (Straus et al., 1996). Therefore, as stated by Marshall et al. (2011), research on IPV “. . .will not progress in an ideal fashion without a better understanding of the reliability of the primary measurement device.” (p. 14)

## Interpartner Agreement Using the Revised Conflict Tactic Scales

The Revised Conflict Tactic Scales (CTS-2; Straus et al., 1996) is probably the most translated and widely used instrument to assess IPV worldwide (Straus & Mickey, 2012). It was created to address criticism of its previous version (e.g., Dobash et al., 1992; Kimmel, 2002) and improve its psychometric properties. The CTS-2 included several changes, such as the addition of scales to assess sexual coercion, injury, and negotiation, the interspersing of the questions’ order, and an increased number of items. Studies conducted across different countries found that this new version has good psychometric properties (e.g., Paiva & Figueiredo, 2006; Straus, 2004; Straus & Mickey, 2012), including stability of self-report of perpetration (Vega & O’Leary, 2007). As with its previous version (Sugarman & Hotaling, 2016), the CTS-2 showed low negative correlations (or non-existent) with social desirability (Bell & Naugle, 2007; Straus, 2004; Straus & Mickey, 2012). Furthermore, asking specific behavioral questions has improved disclosure: Participants have been more likely to recognize the occurrence of a specified behavior than to recognize it as violent (Capinha et al., 2022; Straus et al., 1996). For these reasons, the CTS-2 was the measure chosen for the present work.

The authors of the CTS-2 argued that similar findings were obtained in several studies using husband or wife reports (Straus et al., 1996). However, research on interpartner agreement on the occurrence and frequency of IPV using the CTS-2 has mostly reported the same low-to-moderate interpartner agreement of other instruments, regardless of the perpetrator’s gender. In one notable exception, O’Leary and Williams (2006) found moderate-to-strong agreement levels on physical assault in community samples and focused on the past-year occurrence and frequency. Nevertheless, low agreement levels on sexual coercion and injury subscales were also found. Furthermore, the perpetration of any aggressive behavior and injury was less reported by men and women than victimization by their partners. In another study, Caetano et al. (2009) mostly found low agreement levels on physical assault, psychological aggression, and sexual coercion (injury was not assessed), regardless of the ethnicity of the participants. With regard to differences between gender, women identified themselves as victims and perpetrators of psychological aggression more frequently than men reported them, and men identified themselves as perpetrators of physical assault more often than women reported being victims. Focusing on specific acts (items subscale) of physical assault, Cunradi et al. (2009) identified low agreement levels on male and female perpetration, albeit with a slightly higher agreement on the former. Finally, Marshall et al. (2011) and Graña et al. (2017) investigated agreement on physical assault and psychological aggression and reported low-to-moderate levels, irrespective of gender.

Also using the CTS-2, similar results regarding interpartner agreement have been found in clinical samples (Simpson & Christensen, 2005), including those with couples in which men had alcohol use disorders (Panuzio et al., 2006) or couples in which one of the partners was a war veteran (LaMotte, Taft, Reardon, & Miller, 2014; LaMotte, Taft, Weatherill, et al., 2014).

## Agreement Indexes Considerations

As described earlier, findings about the agreement on the CTS-2 rarely exceed a moderate level. This has allowed continued debates about the findings based on the proxy method. In addition to the reasons that can potentially influence interpartner agreement on IPV mentioned earlier, it may be relevant to consider other methodological issues linked to the indexes themselves, particularly regarding the occurrence. So far, research focusing on interpartner agreement on IPV occurrence has relied mainly on percent agreement and Cohen’s Kappa. Although percentage agreement is an easily interpreted index, it is biased by the occurrence of the behavior and does not account for chance (Simpson & Christensen, 2005). Moreover, the percentage attributed to chance is often unreported. To overcome this limitation, the authors used Cohen’s Kappa concurrently, which is known to correct the level of agreement for chance.

According to some authors (Gwet, 2008; Konstantinidis et al., 2022), some problems arise when using Kappa to assess interpartner agreement on IPV, specifically because it tends to underestimate true variances in small sample sizes and the level of agreement by chance is not known. This leads to high percentages of agreement but low Kappa values, known in the literature as the Kappa paradox (Gwet, 2008; Konstantinidis et al., 2022). Indeed, Kappa was shown to be sensitive to the occurrence of different categories in the population and to differences in the rater’s marginal probabilities (for a complete review of the influence of trait occurrence and marginal homogeneity on inter-rater reliability indexes see Gwet, 2002). This means that, in the presence of a very high or very low occurrence of the assessed behavior or trait, Kappa would not be able to reflect the extent of agreement between raters—it tends to underestimate it (Gwet, 2002, 2008). Furthermore, differences in the results may stem from one category being more commonly observed in one study sample than in others, rather than a true difference in interpartner agreement across studies. These limitations have weakened findings about IPV agreement so far and, once again, impair reliable comparisons between studies (Gwet, 2002; Konstantinidis et al., 2022) or grounded conclusions about using the proxy method in IPV assessment.

To overcome the issues around Cohen’s Kappa, various authors have argued that Gwet’s AC1 should be the statistic of choice, as it outperformed other methods commonly used to assess inter-rater reliability (including Cohen’s Kappa; Dettori & Norvell, 2020; Konstantinidis et al., 2022; Wongpakaran et al., 2013). Indeed, Gwet (2002) demonstrated that AC1 was able to accommodate the behavior prevalence: even with a high or low occurrence of the assessed behavior, AC1 yielded reasonable values and was congruent with the observed values of percent agreement and percent agreement by chance. This happens because the computation of AC1 still considers these parameters but, unlike Cohen’s κ, it reduces the agreement by chance to its correct magnitude, assuming that the propensity for chance agreement is proportional to the portion of ratings that may lead to it (Gwet, 2002; Konstantinidis et al., 2022). Other authors have also argued for the cumulative use of standardized ways of interpreting this agreement index, allowing for reliable comparisons between studies (Dettori & Norvell, 2020; Gwet, 2014).

Research regarding the agreement on IPV frequency has usually resorted to indexes such as Kendall’s Tau-b (e.g., O’Leary & Williams, 2006; Simpson & Christensen, 2005). Tau-b is a measure of the strength of the association between paired observations (Field, 2018; Kendall, 1938). In the context of IPV, it has been used to identify the degree to which the frequency of an aggressive behavior reported by one partner correlates with the frequency of the same behavior reported by the other partner (i.e., the shape of both partners scoring profiles; Furr, 2010). On the other hand, intraclass correlation coefficient (ICC), a widely used reliability index in the literature with couples and families (e.g., Canzi et al., 2019; Silva et al., 2015), has been overlooked in IPV literature. Unlike Tau-b, ICC reflects not only the shape similarity (i.e., the pattern of scores) but also elevation similarity (i.e., the average score across all variables) and scatter similarity (i.e., the variability among the scores; Furr, 2010). Therefore, it represents an absolute index of agreement (i.e., similar profile responses) between two raters (e.g., the partners within a couple) or more, who measure the same target (e.g., the frequency of IPV acts; Furr, 2010; Koo & Li, 2016). As a result, ICC adds information compared to Tau-b, as it can take on low values even in the presence of a positive and significant correlation between reports.

Given these methodological issues and the limited literature available on injury and sexual coercion, it is necessary to conduct additional research on interpartner agreement on perpetration by men and women, using all scales of the CTS-2 and including the agreement indexes discussed above.

## The Present Study

The main goal of this study was to inform IPV assessment practices, namely, the discussion about proxy method *versus* both-partners reports approach for a reliable assessment of IPV. To do so, interpartner agreement on the occurrence and frequency of IPV was assessed in a Portuguese community sample of different-sex couples. Injury and sexual coercion subscales were included in the analyses. The most recent recommendations for the use of agreement indexes were applied. In addition to the usual focus on past-year occurrence and frequency, agreement on occurrence throughout the relationship was also examined. Furthermore, agreement on the negotiation subscale of the CTS-2 was assessed to provide a reference for the comparison of agreement levels.

To the best of our knowledge, this is the first study to analyze interpartner agreement on all CTS-2 subscales using Gwet’s AC1 and ICC and to include the assessment of agreement on occurrence throughout the relationship. It is also the first study to assess interpartner agreement on IPV in Portugal.

Given the characteristics of the AC1 index (Gwet, 2008), higher agreement regarding the different forms of IPV is expected (i.e., moderate to good) compared with those found in previous research using Cohen’s κ. According to previous literature (e.g., Marshall et al., 2011; O’Leary & Williams, 2006), agreement on sexual coercion is expected to be the lowest among the subscales, and agreement on physical assault is the highest. The one-way model of ICC calculated considers the use of a different group of raters (couples), which increases the variety of ratings (i.e., rater effect; Gwet, 2014). Furthermore, ICC considers absolute agreement between partners’ reports rather than their correlation (i.e., shape similarity) only. Therefore, a lower agreement is expected when compared with Tau-b results.

Agreement on occurrence throughout the relationship is expected to be lower than the past-year occurrence for all types of IPV, as some authors have argued that recent events are more easily remembered by partners (Strandmoen et al., 2016). The agreement of frequency is also expected to be lower than the agreement of occurrence, given the added challenge of remembering the exact number of certain behaviors over the past year (Simpson & Christensen, 2005). Agreement levels on the CTS-2 negotiation subscale are expected to be similar to those of the IPV scales, as previously reported (O’Leary & Williams, 2006). Occurrence and frequency were first identified, as less prevalent behaviors are usually associated with lower interpartner agreement (O’Leary & Williams, 2006).

## Method

### Procedures

This study was conducted in accordance with the Declaration of Helsinki. Ethical approval was obtained from the Ethics Committee of the host institution. To be included, participants had to be a partner of different-sex couple, older than 18 years old, married or cohabitating for at least 3 months, and with no self-reported psychotic disorder or symptoms. Both members of the couple must agree to participate and at least one of them must be Portuguese. If one of them was not, he or she must speak Portuguese fluently. The sample was non-probabilistic and recruited through a snowball method, both in urban and in rural areas. After being informed about the goals of the study and the confidentiality and anonymity of the data, all participants provided oral and written informed consent for their participation. All participants completed the CTS-2, in addition to measures that comprised a research protocol not relevant to the present study. Questionnaires were delivered in separate envelopes, and couples were clearly instructed to respond to them privately, independently, and without cooperation. All data exclusions, all manipulations, and all measures in the study are reported.

### Participants

Couples in this sample (*N* = 268 couples) were aged between 21 and 81 years old (*M* = 43.75, *SD* = 11.80; *M_men_* = 44.67; *SD =* 11.92; *M_women_* = 42.82; *SD* = 11.63). Most couples (69.4%) were married, and 68.3% reported having one or two children (with 23% reporting no children). The average relationship length was 11.42 years (*SD* = 11.83). Most men (50.6%) and women (58.1%) had a college education, were employed (88.1% and 83.7%), and did not consider themselves financially dependent on their partner (86.5% and 80.1%). Most of these couples lived in an urban area (66.6%), and only 2.6% of the participants did not have Portuguese nationality. Of the total sample, 40 couples (16.4%) reported no history of violence during their relationship, and 81 (30.2%) reported no history of violence during the 12 months prior to the study.

### Measure

The Conflict Tactic Scales-Revised (CTS-2) (Straus et al., 1996; Portuguese version by Paiva & Figueiredo, 2006) was used. It is a 78-item self-report questionnaire measuring physical assault, psychological aggression, injury, sexual coercion, and negotiation, within the couple. Using an eight-point scale ranging from (1) “Once in the last year” to (6) “More than 20 times in the last year,” including the options (7) “Not in the last year but have occurred previously,” and (8) “Never occurred,” respondents are asked to rate whether, and how often, they (perpetration) or their partner (victimization) had engaged in the behaviors described. Scores on the items were dichotomized to assess the past-year occurrence (i.e., scores of 1–6) and occurrence throughout the relationship (i.e., relationship occurrence; scores of 1–7). Frequency was assessed by evaluating the number of incidents of violent or aggressive acts reported at least once in the past year (calculated using the midpoint, as recommended by Straus et al., 1996). In the present study, Cronbach’s alphas of CTS-2 subscales were: .64 and .67 for sexual coercion victimization and perpetration, .76 and .78 for psychological aggression perpetration and victimization, .79 for both scales of negotiation, .93 and .94 for injury perpetration and victimization, and .96 and .97 for physical assault perpetration and victimization.

### Analytical Procedures

According to previous guidelines (see Armstrong et al., 2002), different agreement indexes are presented, as they can lead to different conclusions. IBM SPSS STATISTIC 22 was used to compute descriptive statistics, mean and proportion comparisons, Cronbach’s alphas, and Tau-b correlations. RStudio (Version 1.4.1717) was used to handle missing values and to compute Gwet’s AC1 and percent agreement indexes (irrCAC Package) as well as ICC estimates and their 95% confidence intervals (CIs; using the irr Package, one-way random effects model). Four couples in which at least one member had more than 50% missing responses on the research protocol were excluded from the sample. Missing data (1.14%) were imputed using multivariate imputation by chained equations (MICE; van Buuren & Groothuis-Oudshoorn, 2011), under the random forest algorithm (Shah et al., 2014), with 10 multiple imputations, and 50 maximum imputations. Random forest-based MICE algorithm reduces the risk of overfitting by resorting to bootstrap aggregation of multiple regression trees and combining many predictions to create a more accurate one (it is considered non-deterministic; Shah et al., 2014). It aims to overcome problems associated with parametric settings of MICE implementation (namely, the omission of important nonlinear terms, not including more predictor variables than the number of observations without resorting to prior information, and collinearity problems due to the inclusion of highly correlated variables; Hardt et al., 2012; Seaman et al., 2012; Zhao & Long, 2016). Density plots showed that imputed data followed the same distribution as the original data.

AC1, percent agreement, and percent chance agreement were reported as measures of interpartner agreement on the occurrence of IPV (i.e., based on categorical variables). The interpretation of AC1 was based on Altman’s benchmark scale presented by Gwet (2014; i.e., <.20 = *poor*; .21 to .40 = *fair*; .41 to .60 = *moderate*; .61 to .80 = *good*; .81 to 1.00 = *very good*), using the standardized method of benchmarking proposed by the author. Through this method, one can calculate the benchmark range membership probability based on the index value and the standard error associated. These probabilities are then added from the higher range to the lowest resulting in the cumulative probability (CumProb) of an agreement coefficient falling within a given benchmark range. A threshold of .95 was defined according to Gwet’s (2014) guidelines. The first benchmark range associated with a CumProb equal to or higher than .95 is used to interpret the AC1 index. This method prevents misleading conclusions from using any benchmark scale alone, as it does not depend on sample size nor the distribution of occurrence among categories. For these reasons, it allows for comparisons between studies that follow this methodology (Dettori & Norvell, 2020; Gwet, 2014).

Kendall’s Tau-b and ICC were computed as indexes of interpartner agreement on the frequency of violent behaviors (i.e., based on continuous variable). Tau-b values measure the strength of associations between variables and range between −1 (perfect disagreement) and 1 (perfect agreement; Field, 2018; Kendall, 1938). Its interpretation followed the guidelines proposed by Botsch (2011; i.e., < .10 = *very weak*; .10 to .19 = *weak*; .20 to .29 = *moderate*; ≥ .30 = *strong*, regardless of the direction of the relationship). ICC, in turn, is calculated based on mean squares obtained through analysis of variance. It ranges from 0 to 1, with values close to 1 representing higher agreement (Koo & Li, 2016). ICC interpretation followed the Koo and Li (2016) guidelines (i.e., < .50 = *poor*; .50 to .75 = *moderate*; .75 to .90 = *good*; > .90 = *excellent reliability*). As some authors argued that including non-aggressive/non-violent couples could inflate the agreement on frequency (Graña et al., 2017; Marshall et al., 2011; Panuzio et al., 2006), index agreements were computed for the complete sample but also for a subsample excluding couples where no violence was reported in the past year by either partner. This subsample was named “IPV couples” for an easier distinction from this point forward. Nonetheless, this expression should not be interpreted as more than a designation for those couples who reported the use of at least one type of IPV behavior in the past 12 months.

## Results

### Past-Year and Relationship Occurrence, and Past-Year Frequency, of Different Types of IPV and Negotiation Strategies Reported by Men and Women

Past-year and relationship occurrence of all types of IPV and negotiation strategies were reported regarding perpetration and victimization by men and by women (cf. Table 1). Whether considering the reports of men or women, the most reported form of IPV was psychological aggression, ranging from 48.1% (men’s past-year victimization) to 66.8% (women’s perpetration throughout the relationship). The least reported type of IPV was injury, ranging from 1.9% (men’s past-year perpetration) to 4.5% (women’s victimization throughout the relationship). Both men and women reported having perpetrated more physical assault against their partner than having suffered physical assault (victimization) in the past year (7.5% vs. 6.7% for men, and 10.8% vs. 6.8% for women) and throughout the relationship (14.9% vs. 13.4% for men and 18.7% vs. 14.9% for women). Perpetration of sexual coercion by men was more reported by men and women (16.8% and 14.2% in the past year, and 23.9% and 23.5% throughout the relationship).

**Table 1.** Past Year and Relationship Occurrence of Men’s and Women’s Reports of Perpetration, Victimization, and Negotiation by Gender.

Type of violence	Men’s reports		Women’s reports	
Past year	P (%)	V (%)	V (%)	P (%)
Physical assault	7.5	6.7	8.6	10.8
Psychological aggression	49.6	48.1	50.4	49.6
Sexual coercion	16.8	11.9	14.2	9.3
Injury	1.9	2.6	2.6	2.6
Adaptive strategies	MU (%)	WU (%)	MU (%)	WU (%)
Negotiation	94.4	94.4	94.0	94.0
Type of violence				
Throughout relationship	P (%)	V (%)	V (%)	P (%)
Physical assault	14.9	13.4	14.9	18.7
Psychological aggression	65.3	63.4	66.0	66.8
Sexual coercion	23.9	15.3	23.5	14.6
Injury	3.7	3.4	4.5	3.4
Adaptive strategies	MU (%)	WU (%)	MU (%)	WU (%)
Negotiation	98.5	98.1	98.9	98.9

*Note*. P = perpetration; V = victimization; MU = men's use; WU = women's use.

The frequency of all types of IPV and negotiation strategies were also reported regarding men’s and women’s perpetration or use (cf. Table 2). Men tended to report a higher frequency of perpetration and victimization than women. Psychological aggression was also the most frequent type of IPV reported, whether considering all the sample (ranging from 5.88 for perpetration by women to 7.42 for victimization by men) or the IPV couples only (ranging from 8.42 perpetration reported by men to 10.64 for victimization by men).

**Table 2.** Past-Year Frequency by Types of Violence and Negotiation in All Couples (N = 268) and IPV Couples (n = 187).

Type of violence	Men’s reports		Women’s reports	
All couples	P	V	V	P
(*N* = 268)	*M (SD)*	*M (SD)*	*M (SD)*	*M (SD)*
Physical assault	2.35 (19.39)	2.02 (18.28)	0.94 (8.51)	0.93 (6.66)
Psychological aggression	6.58 (17.86)	7.42 (19.41)	6.49 (13.47)	5.88 (10.61)
Sexual coercion	3.36 (13.41)	2.70 (12.67)	2.59 (8.03)	1.86 (7.13)
Injury	1.05 (10.20)	1.09 (10.36)	0.51 (4.71)	0.46 (4.05)
Adaptive strategies	MU	WU	MU	WU
Negotiation	58.73 (38.37)	57.28 (37.57)	57.96 (37.22)	59.36 (37.40)
IPV couples	P	V	V	P
(*n* = 187)	*M (SD)*	*M (SD)*	*M (SD)*	*M (SD)*
Physical assault	3.36 (23.16)	2.90 (21.84)	1.35 (10.17)	1.33 (7.95)
Psychological aggression	9.43 (20.76)	10.64 (22.51)	9.29 (15.30)	8.42 (11.83)
Sexual coercion	4.81 (15.85)	3.87 (15.03)	3.71 (9.40)	2.67 (8.42)
Injury	1.50 (12.20)	1.57 (12.39)	0.73 (5.62)	0.66 (4.84)
Adaptive strategies	MU	WU	MU	WU
Negotiation	62.64 (36.23)	60.68 (35.47)	60.11 (32.91)	62.25 (32.85)

*Note*. IPV = intimate partner violence; P = perpetration; V = victimization; SD = standard deviation; MU = men's use; WU = women's use.

### Interpartner Agreement on the Occurrence of Different Types of IPV and Negotiation During the Past-Year and Throughout the Relationship

Regarding the occurrence of IPV (past-year and throughout the relationship) (cf. Table 3), the percent agreement identified was higher than the value expected by chance for all types of IPV perpetrated by men and women. The same was true for the report of negotiation used by any partner. Relying on AC1, agreement ranged from moderate (AC1 = .53, *p* < .001) to very good (AC1 = .97, *p* < .001) in all forms of violence, except for the past-year occurrence of psychological aggression, which was only fair both for the perpetration by men (AC1 = .48, *p* < .001) and women (AC1 = .48, *p* < .001). Notably, the agreement regarding the perpetration by men and women was very similar in all forms of violence, except for sexual coercion throughout the relationship, for which the agreement was higher for women’s perpetration (AC1 = .77, *p* < .001) than for men’s perpetration (AC1 = .66, *p* < .001).

**Table 3.** Interpartner Agreement on the Occurrence of Different Types of IPV and Negotiation During the Past Year and Throughout the Relationship.

Past year occurrence	Percent agreement		Percent chance agreement		AC1 (*SE*)			
	Men P	Women P	Men P	Women P	Men P	Range (CumProb)	Women P	Range (CumProb)
Physical assault	.89	.88	.14	.16	.87*** (.02)	Very good (1.00)	.86*** (.03)	Very good (.99)
Psychological aggression	.74	.74	.50	.50	.48*** (.05)	Fair (1.00)	.48*** (.05)	Fair (1.00)
Sexual coercion	.82	.87	.26	.19	.76*** (.04)	Good (1.00)	.84*** (.03)	Good (1.00)
Injury	.97	.96	.04	.05	.97*** (.01)	Very good (1.00)	.96*** (.01)	Very good (1.00)
Adaptive strategies	MU	WU	MU	WU	MU		WU	
Negotiation	.95	.95	.11	.11	.95*** (.02)	Very good (1.00)	.95*** (.02)	Very good (1.00)
Throughout relationship occurrence	Men P	Women P	Men P	Women P	Men P		Women P	
Physical assault	.83	.84	.25	.27	.77*** (.04)	Good (1.00)	.78*** (.04)	Good (1.00)
Psychological aggression	.75	.74	.45	.45	.55*** (.05)	Moderate (1.00)	.53*** (.05)	Moderate (.99)
Sexual coercion	.78	.83	.36	.25	.66*** (.05)	Moderate (1.00)	.77*** (.04)	Good (1.00)
Injury	.96	.96	.08	.06	.96*** (.01)	Very good (1.00)	.96*** (.01)	Very good (1.00)
Adaptive strategies	MU	WU	MU	WU	MU		WU	
Negotiation	.99	.99	.03	.03	.99 ***(.001)	Very good (1.00)	.98*** (.01)	Very good (1.00)

*Note.* Men P = men’s perpetration; Women P = women’s perpetration; CumProb = cumulative probability of an agreement; SE = standard error; MU = men’s use; WU = women’s use.

*** *p* < .001.

The standardized method used to interpret AC1 agreement index (i.e., using the first benchmark range that has a CumProb equal or higher than .95 of being associated with the AC1 value) led to the same interpretation of results as a more straightforward method would (i.e., direct comparison of the index value with Altman’s benchmark scale ranges). Exceptions were the agreement on past-year occurrence of psychological aggression perpetrated by men (CumProb for Moderate range = .93) and by women (CumProb in Moderate range = .93), on past year sexual coercion perpetrated by women (CumProb in Very good range = .91), and in occurrence throughout the relationship of sexual occurrence perpetrated by men (CumProb in Good range = .88).

### Interpartner Agreement on the Frequency of Different Types of IPV During the Past Year

Regarding the frequency of different types of IPV (cf. Table 4), ICC pointed to a poor level of agreement (< .50) for all the perpetration forms by men and women, for both the complete sample and for the subsample of couples that reported at least one type of IPV during the past year (IPV couples; *n* = 187). The agreement levels considering Tau-b were strong (≥ .30) for most types of IPV perpetration by men and women in the complete sample. Exceptions were the perpetration of physical assault by men (Tau-b = .28) and women (Tau-b = .29), and the perpetration of injury by women (Tau-b = .26), all showing an agreement level deemed as moderate. Considering the subsample of IPV couples, agreement levels based on Tau-b were identified as moderate for the perpetration of all IPV types by men and women, except for the perpetration of injury by men (in which a strong agreement of .32 was found).

**Table 4.** Interpartner Agreement on the Frequency of Different Types of IPV During the Past-Year.

All couples (*N* = 268)	ICC				Tau-b correlation	
Type of violence	Men's perpetration	95% CI	Women's perpetration	95% CI	Men's perpetration	Women's perpetration
Physical assault	.23	[.11, .34]	.22	[.11, .33]	.28***	.29***
Psychological aggression	.32	[.21, .42]	.33	[.22, .44]	.47***	.46***
Sexual coercion	.25	[.14, .36]	.25	[.13, .36]	.34***	.32***
Injury	.27	[.15, .38]	.23	[.12, .34]	.33***	.27***
Adaptive strategies	Men's use	95% CI	Women's use	95% CI	Men's use	Women's use
Negotiation	.52	[.43, .61]	.52	[.43, .61]	.37***	.38***
IPV couples (*n* = 187)	ICC				Tau-b correlation	
Type of violence	Men's perpetration	95% CI	Women's perpetration	95% CI	Men's perpetration	Women's perpetration
Physical assault	.23	[.09, .36]	.22	[.08, .35]	.25***	.27***
Psychological aggression	.26	[.13, .39]	.28	[.14, .41]	.29***	.27***
Sexual coercion	.23	[.09, .36]	.23	[.09, .36]	.29***	.29***
Injury	.27	[.13, .39]	.23	[.09, .36]	.32***	.26***
Adaptive strategies	Men's use	95% CI	Women's use	95% CI	Men's use	Women's use
Negotiation	.46	[.33, .56]	.45	[.33, .56]	.32***	.32***

*Note.* IPV = intimate partner violence; ICC = intraclass correlation coefficient; CI = confidence interval.

*** *p* < .001.

Results of agreement, based on ICC about the frequency of negotiation strategies used by men and women in the past year, revealed moderate levels of agreement in the complete sample (ICC = .52 and ICC = .53, respectively). Agreement levels drop to poor (namely, to ICC = .46 and ICC = .45, although the CIs included the moderate range) restricting the analysis to couples that reported at least one type of IPV during this period. Based on Tau-b correlations, agreement levels regarding the frequency of negotiation strategies used by men and women were strong (Tau-b = .37 and Tau-b = .38) in the complete sample. IPV couples only showed moderate agreement (Tau-b = .32, for both men and women use) regarding this variable.

## Discussion

Both practitioners and researchers need to be confident that the measures they are using are robust and valid. This is especially important when dealing with sensitive and pervasive phenomena with intergenerational consequences such as IPV. Because the proxy method is used frequently in the context of IPV research, it is important to explore whether it is a reliable approach to assess IPV frequency and occurrence. It is assumed that the higher the agreement between the partners’ reports on IPV, the higher the reliability of the proxy method to assess it. Therefore, this work aims to inform assessment practices in IPV targeting different-sex couples in community settings.

As there is no prior research on interpartner agreement regarding IPV in Portugal, this first study focuses on couples (different-sex) that are easier to access to collect data (Capinha et al., 2022). The agreement about different types of IPV is investigated, following the most recent recommendations regarding inter-rater reliability to overcome Cohen’s Kappa limitations (Dettori & Norvell, 2020; Gwet, 2014; Konstantinidis et al., 2022; Wongpakaran et al., 2013). Other reliability indexes deemed suitable to assess interpartner agreement on IPV frequency (not only occurrence) are also included, and agreement on IPV occurrence throughout the relationship is assessed. Both men’s and women’s perpetration are analyzed. Sexual coercion and injury are included in the analyses, in addition to the most extensively studied physical assault and psychological aggression. As research focusing on these forms of IPV is scant, this work provides relevant data regarding interpartner agreement in their report. This work further includes the analysis of agreement levels on negotiation use as a strategy to cope with conflict, a subscale of CTS-2 usually omitted in agreement analysis.

Findings show that women tend to report the occurrence of IPV slightly more, whether perpetrated by men (victimization) or by themselves (perpetration), when compared with men. Sexual coercion is the exception. This type of IPV is more reported by men, both as perpetrators and as victims. In contrast, men tend to report themselves as having perpetrated or suffered more frequent acts of IPV than women. Nonetheless, this study fails to find any pattern of agreement associated with gender or the role of perpetrator *versus* victim, as agreement levels were similar across gender, whomever the perpetrator was. This is in accordance with previous research in which no associations between gender and levels of agreement were found (Marshall et al., 2011; Moffitt et al., 1997).

As hypothesized, AC1 yields mainly moderate to good agreement levels, higher than those usually identified in the literature. This indicates that Cohen’s Kappa paradox may have led to an underestimation of agreement in IPV reports in past research. In this regard, it is worth noticing that AC1 values are in accordance with the percentages of agreement and agreement by chance identified in this sample. This accordance supports using AC1 as a proper agreement index regarding the occurrence of IPV. Moreover, the use of the standardized method of interpreting AC1 index appears to be appropriate: it allows obtaining an interpretation different from the classic method (that is, the direct comparison between the agreement value and the benchmark range) and comparing it with those of other studies using the same method. To allow these comparisons to be made, future research should report the AC1 values and standard errors, as well as cumulative probabilities of agreement.

Still considering IPV occurrence, the agreement throughout the relationship was found to be lower than past-year agreements. The injury scale is the most consensual, both for past-year and throughout the relationship, followed by physical assault. These are the subscales with the more objective items, which probably help identify whether a specific action has happened or not (O’Leary & Williams, 2006). The suggestion that less objective items (i.e., those from sexual coercion or psychological aggression subscales) are more prone to subjective interpretation and could even depend upon the attribution of the behavior intention has been previously advanced (Caetano et al., 2009; Simpson & Christensen, 2005). Such an argument could help to explain the findings regarding psychological aggression. Despite its higher occurrence and contrary to what was expected, psychological aggression is the scale with the lowest agreement levels on occurrence in the past year and throughout the relationship (either relying on the percentage or AC1).

Regarding interpartner agreement on IPV frequency, as expected, findings show that agreement estimations based on ICC yield lower levels than those based on Tau-b. Across all couples, both indices show that psychological aggression is the scale with a higher agreement regarding its frequency. This finding shows that even if partners have different reports regarding the occurrence of psychological aggression, they tend to agree on whether it is a frequent behavior. For the subsample of couples reporting violence in the past year (i.e., IPV couples), the agreement on the perpetration of psychological aggression by men based on ICC is quite similar to the agreement on perpetration of injury by them (with the same CI). According to Tau-b, it is the perpetration of injury by men and the perpetration of sexual coercion by women that gather the highest agreement levels on frequency in this subsample. These findings indicate that it is easier for partners within IPV couples to agree on whether sexual coercion perpetrated by women is frequent rather than how frequent it is. On the contrary, injury and psychological aggression perpetrated by men tend to be those IPV types in which partners’ reports vary in the same direction (shape similarity, as assessed by Tau-b) and are also more consistent in the specific frequency of identified behaviors (absolute agreement, as assessed by ICC). Given that IPV couples are those who reported at least one IPV behavior in the past year, the prevalence of IPV in this sample may influence the frequency agreement in different ways (O’Leary & Williams, 2006). It is important to note that both indices indicate that agreement on frequency tends to be lower in this subsample of couples. Nonetheless, the decrease of agreement seems to be higher regarding the answer’s pattern similarity (Tau-b) than regarding the absolute agreement (ICC). This could be happening due to an increase in the average similarity (i.e., a smaller difference between the means of IPV frequency reported by each partner) or scatter similarity (i.e., a lower discrepancy between the variance of both partners' answers; Furr, 2010). Such a hypothesis would imply that in settings with a higher prevalence of IPV behaviors, partners tend to report IPV frequency in a more cohesive fashion, even if they agree less on which particular behaviors were perpetrated more or less frequently.

The divergences in the agreement levels between samples with different IPV occurrence rates support the recommendation of excluding non-aggressive couples from the analyses (e.g., Graña et al., 2017; Marshall et al., 2011; Panuzio et al., 2006) to obtain more conservative estimates of the agreement on IPV frequency and occurrence. By doing so, agreement levels are not inflated by the results of those couples that agree on the non-occurrence (therefore, no frequency) of IPV. Nonetheless, this would depend on the purpose of measuring the agreement. If one wants to evaluate whether the proxy method is reliable to estimate prevalence rates, one should also consider whether couples agree on the non-occurrence of IPV. Researchers (e.g., Graña et al., 2017; Marshall et al., 2011) have also stressed the need to investigate interpartner agreement in forensic samples (where a higher occurrence of violence is expected). Legal and forensic settings could introduce critical contextual factors that inhibit any extrapolation of the conclusions based on community samples.

The hypothesis that the agreement on the frequency would be lower than the agreement on occurrence holds true if it is comparing the frequency with absolute agreement on frequency. Indeed, according to Koo and Li (2016) guidelines the ICC estimates that were found are deemed as poor. Conversely, Tau-b pointed to moderate-to-strong interpartner agreement. Thus, agreement on frequency based on the shape similarity of both partners answers’ patterns would be analogous to the agreement on occurrence (mainly identified as moderate-to-very good). Similarly, the verification of the hypothesis regarding the agreement in IPV scales being in the same range of negotiation agreement levels also depends on the chosen index. If compared with the highest agreement on past-year occurrence and occurrence throughout the relationship (i.e., injury), negotiation presents a similar percent and percent by chance agreements. It also presents AC1 values in the same range (very good). This seems to allow for the interpretation that partners within different-sex couples in the community tend to agree on their report about the occurrence of IPV around the same they agree on the occurrence of other (more benevolent) behaviors within the intimate relationship. Regarding frequency, agreement on negotiation is only comparable to the highest agreement based on Tau-b, but not on ICC. Hence, partners within different-sex couples seem to be likewise able to agree on whether IPV behavior or negotiation is frequent. Nevertheless, they also seem to agree on how often they or their partner have used negotiation more than they agree on how often they used IPV.

The above findings underline the importance of using different index agreements, as different estimates may be reached when they are applied to the same data (Armstrong et al., 2002; O’Leary & Williams, 2006). Additional research must be done to confirm the consistency of these findings using the same agreement indexes, particularly because the agreement levels identified in this study are generally higher than those found in the literature (e.g., Caetano et al., 2009; Cunradi et al., 2009; LaMotte, Taft, Reardon, & Miller, 2014). Even so, regarding interpartner agreement on the occurrence of most types of IPV, findings show that reports of different partners tend to yield similar results. However, the data become more difficult to interpret concerning the agreement on the frequency as the indexes used lead to different conclusions. Indeed, different agreement levels based on different indexes and for different types of violence found in this study indicates the suitability of proxy method to assess IPV occurrence (i.e., prevalence) but do not give evidence for the validity of the proxy method regarding IPV frequency. This means that findings about bidirectionality and symmetry, which are mainly based on self-report of IPV occurrence by one partner only (Esquivel-Santoveña et al., 2013; Langhinrichsen-Rohling et al., 2012), would be reliable. Given the advantages of this method (Moffitt et al., 1997; Straus et al., 1996), its use might be the best option for large-scale or epidemiologic studies, especially if a descriptive perspective is intended.

It is important to consider that obtaining full (or even very high) agreement in IPV reports is probably impossible, at least using self-report instruments that are subject to measurement error (Moffitt et al., 1997). Furthermore, as Simpson and Christensen (2005) argue, IPV assessment seems to focus on the perception of each partner, and “there may be no real “truth”” (p. 430) to be evaluated. This is probably why most authors (e.g., O’Leary & Williams, 2006; Yoshikawa et al., 2021), including the ones of the present study, continue to recommend collecting both partners’ reports. From a clinician’s perspective, this strategy can be useful to explore the reasons underlying inconsistencies between partners’ reports during the psychotherapeutic process. From a researcher’s perspective, analyzing both partners’ reports would increase the understanding of how the couple interacts.

Even when it is possible to collect both partners’ reports, questions arise on how to solve the problem posed by their inconsistencies. It is often assumed that taking the highest report within the couple (called the upper-bound estimate) is the best solution (Armstrong et al., 2002; O’Leary & Williams, 2006; Straus et al., 1996). However, that may not always be the case as there is no way to guarantee that the highest report is the most reliable. Both men and women could be prone to over or underreport aggressive behavior due to different factors (e.g., social desirability, memory, education, fear, shame, self-justification, and relationship satisfaction; Armstrong et al., 2002; Marshall et al., 2011; Yoshikawa et al., 2021). Chances for over or underreporting IPV are present, even considering a lower probability of participants intentionally manipulating their report of IPV in research focusing on community samples (Moffitt et al., 1997). If research relies on the upper-bound estimates, this may lead to overestimating the magnitude or severity of the phenomenon. Conversely, relying on the lowest of the partners’ reports or requiring perfect interpartner agreement to consider that an IPV event occurred also has disadvantages: it may lead to missing a substantial number of events and underestimating the IPV occurrence or severity (Caetano et al., 2009). Therefore, this strategy is also not appropriate. In other words, gathering information from both partners is not a panacea. If not properly dealt with, it can also hinder decision-making in clinical settings, misguide public policies, or impair the appropriate distribution of resources to tackle IPV.

To avoid misusing both partners’ information and maximize its potential in understanding IPV, resorting to statistical techniques that accommodate the variations and interactions in partner reports should be prioritized. Good examples are the Actor and Partner Interdependence Model (APIM; Kenny et al., 2006) or other multilevel analyses (as used by Marshall et al., 2011; Graña et al., 2017) which allow for differences in partners’ reports of the same information. This would improve the integration of both partners’ views into a more complete and coherent understanding of the behaviors and processes in IPV, a need emphasized by several authors (Graña et al., 2017; Marshall et al., 2011; Simpson & Christensen, 2005). Future studies should also investigate possible correlates of agreement at an individual and dyadic level (e.g., marital satisfaction, education, and relationship length), as existing findings/studies are not consistent (e.g., Graña et al., 2017; Marshall et al., 2011; Simpson & Christensen, 2005). Furthermore, future research should consider replicating this study’s findings in samples with lower levels of education. Education has been identified as a factor that may influence interpartner agreement (Armstrong et al., 2002; Yoshikawa et al., 2021). Most of the sample having a college education may have contributed to a better (and more homogeneous) understanding of the questions, leading to a higher interpartner agreement in this study. Finally, new research should include all CTS-2 scales, particularly injury, as it was integrated into the instrument to better understand the consequences of the reported behaviors.

When interpreting the findings of this study, it is important to bear in mind that these refer to reports on the CTS-2. The CTS-2 does not include the assessment of severe sexual violence (e.g., rape, near-lethal violence) or severe coercive and controlling behaviors (e.g., social deprivation). Other instruments, using different item formulations and exploring the presence of other aggressive acts could yield different findings. Nonetheless, recent findings (e.g., Riesgo González et al., 2019; Marshall et al., 2021) have pointed to limited agreement between partners in other measurements as well. This again suggests that much higher agreement may not be possible regardless of the instrument used.

The current study is not without limitations, which should also be acknowledged in the interpretation of its findings. First, the use of a non-probabilistic sample that only includes different-sex couples impairs the generalization of the findings to the broader population. Second, it is not possible to guarantee that each member of a couple was able to ensure his or her privacy when responding to the questionnaire, despite the clear instructions to respond privately, independently, and without cooperation, which may have influenced their report. Third, the already mentioned issue of scales’ reliability reinforces the need for more research with larger samples. In addition, this study does not allow testing for reasons why interpartner agreement is not high on all IPV types, nor to identify whether the most reliable reports are those from men or women. Therefore, future research should (re)visit these questions, as evidence on correlates and predictors of agreement is not coherent and could be useful to ascertain conditions for a more reliable assessment. Future studies should also try to replicate these findings in different settings (e.g., legal settings) and samples (e.g., including non-binary and same-sex couples), as research concerning these populations is scarce and points to low interpartner agreement (Stephenson et al., 2019; Walsh & Stephenson, 2022).

Nonetheless, despite these limitations, this study contributes to advancing current knowledge on inter-rater reliability in the IPV reports, by including agreement indexes that have not been used in these analyses and which overcome some of the limitations of more traditional ones. It also goes beyond existing research by including the assessment of all forms of IPV frequency and occurrence in the past year and throughout the relationship. The levels of agreement found corroborate that the CTS-2 is relevant and, at least, as reliable as other instruments to assess IPV in community samples. Finally, this study discusses and critically reflects on the standard practices on IPV assessment and challenges researchers and practitioners to do so. Although the proxy method may be adequate in some contexts (as supported by this study’s findings), full agreement is likely impossible to reach. Furthermore, collecting reports from both partners enriches the available information. However, it is critical that the statistical analysis of this information considers both reports equally, as well as their mutual influences. Thus, evaluating both partners, followed by dyadic analyses, should become standard practice in future IPV research.
